# Herpes Encephalitis Masquerading as Tumor

**DOI:** 10.5402/2011/474672

**Published:** 2011-04-19

**Authors:** Tasneem Peeraully, Joseph C. Landolfi

**Affiliations:** New Jersey Neuroscience Institute, John F. Kennedy Medical Center, 65 James Street, Edison, NJ 08820 -3947, USA

## Abstract

A 54 year old lady presented with lethargy and 15 kg weight loss over the past year. CT scan of the head revealed left temporal lobe hypodensity with a discrete area of hemorrhage within the left mesial temporal lobe. Due to concerns about impending central herniation, lumbar puncture was not performed. MRI of the brain showed a large lesion of the left temporal lobe, extending to the left frontal lobe, and very patchy meningeal enhancement. There was a noncontiguous lesion of the right insula. A differential diagnosis of herpes simplex encephalitis (HSE) and multifocal infiltrative glioma was entertained. MR spectroscopy demonstrated an increased choline peak at the level of the medial left temporal lobe and MR perfusion demonstrated patchy areas of hyperperfusion within the left anterior temporal lobe, both suggestive of neoplastic disease. Following open brain biopsy, pathology revealed herpes simplex virus (HSV) positive nuclei in the cortex and subcortical white matter. As both herpes simplex encephalitis and low-grade glioma demontrate MRI findings of hypointensity on T1 images and hyperintensity on T2 images, the diagnosis of herpes encephalitis can be clouded by confounding factors, especially when cerebrospinal fluid (CSF) cannot be obtained.

## 1. Introduction

Herpes simplex encephalitis has an incidence of 1 in 250,000 to 500,000 persons per year and is the most common sporadic fatal encephalitis in the Western world [1]. It typically presents with headache, fever, and confusion developing over hours to days. Focal neurological findings including hemiparesis, cranial nerve deficits, visual field loss, and dysphasia. Focal or generalized seizures may also be seen [2]. Patients can exhibit behavioral changes with personality changes and psychosis. 

CT head imaging shows hypodense lesions of the temporal lobe and orbitofrontal regions and may demonstrate mass effect. Petechial hemorrhage may not always be observed on CT [3, 4]. T2-weighted MRI reveals hyperintensity corresponding to edematous changes in the temporal lobes, characteristically sparing the basal ganglia. T1-weighted images show hypointense signal in the affected areas, and meningeal enhancement may be demonstrated following the administration of gadolinium [5].

When CSF can be obtained, it shows mononuclear cell pleocytosis in 97% of cases, typically with a mildly elevated protein and a normal glucose [2]. Polymerase chain reaction (PCR) assays performed on specimens from patients with brain biopsy-proven herpes simplex encephalitis reveal a diagnostic sensitivity of 98% at the time of clinical presentation as well as a specificity approaching 100% [6]. Of note, negative PCR assay for HSV DNA on the first or second day of illness may become positive on testing of a subsequent CSF specimen [7].

Gliomas also demonstrate MRI findings of hypointensity on T1 images and hyperintensity on T2 images. Discrete hemorrhage is more typically seen in tumor than HSE. Gliomas can be focal, diffuse, or multifocal. Though usually absent, minimal enhancement can occasionally be seen in glioma. The clinical features of viral encephalitis, namely headache, fever, seizures, and encephalopathy, may be seen in patients with high-grade gliomas [8]. In cases where imaging is ambiguous and CSF is not available, biopsy may ultimately be needed to make a diagnosis.

## 2. Case Report

A 54-year-old female was evaluated for altered mental status in the emergency room. Eight days prior, the patient had complained of headache and facial tenderness. She had been feeling drowsy and unwell, but she had not been febrile. On the day she was taken to the emergency room, her sister had found her lethargic and verbalizing poorly. The patient had been having intermittent headaches for the past year associated with nausea and vomiting. She had also had 15 kg weight loss over 1 year.

**Figure 1:**
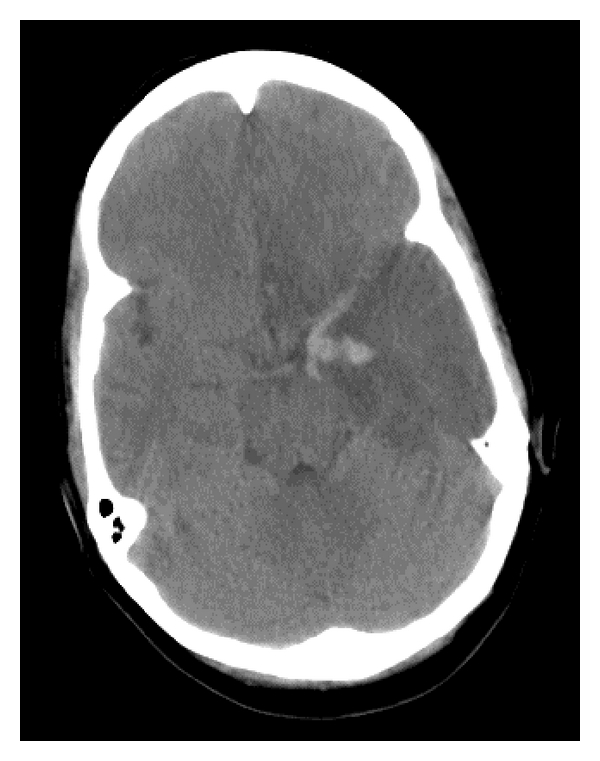
CT head: left temporal lobe hypodensity with a discrete area of hemorrhage.

Initially, the patient was oriented to person and place although lethargic, not communicating well, with slurred speech, poor eye contact, and exhibiting decreased psychomotor activity. Her pupils were 3 mm and reactive bilaterally. Neurological examination was nonfocal except for brisk reflexes in the right upper extremity. Blood work did not reveal any leukocytosis (white cell count was 6.5 thousand/*μ*L) or electrolyte imbalance. Erythrocyte sedimentation rate was 67 mm/hour, and CRP was 7.74 mg/dL. Urine toxicology screening was negative. A CT scan of the head showed left temporal lobe hypodensity, 8.3 centimeters in greatest diameter, with areas of hemorrhage within mesial left temporal lobe (see Figure 1). A lumbar puncture was not performed because of concerns regarding herniation. The patient was placed on dexamethasone and acyclovir. Levetiracetam was started as it was not clear that the patient whether seizure was responsible for her acute presentation.

**Figure 2:**
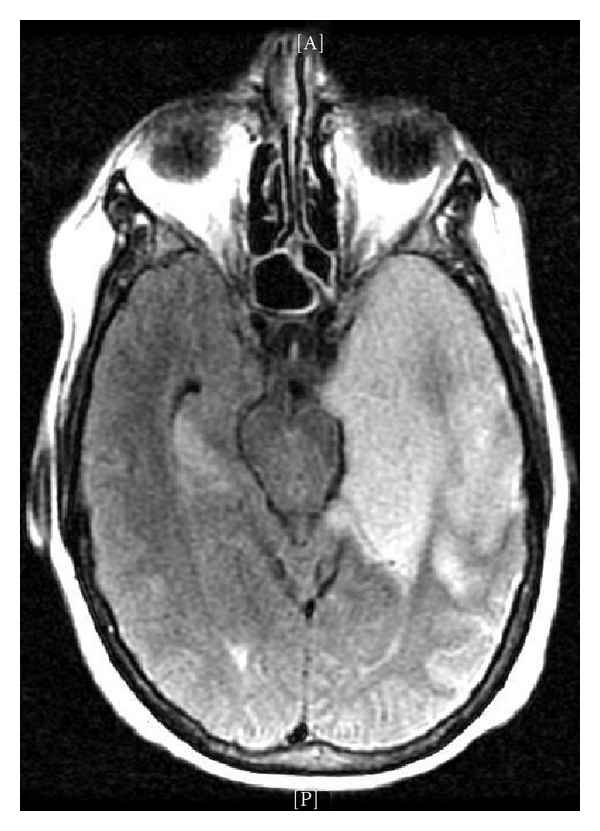
MRI T2 FLAIR: hyperintensity of lesions on T2.

**Figure 3:**
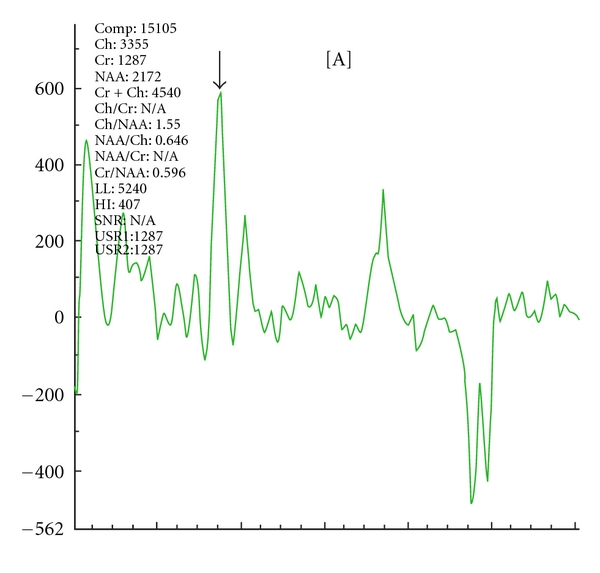
MR spectroscopy: choline peak (as indicated by arrow) at the level of the medial left temporal lobe.

MRI of the brain was obtained, showing a large lesion of the left temporal lobe with extension to the frontal lobe and noncontiguous ill-defined lesions of the right temporal lobe (see Figure 2). A differential diagnosis included herpes encephalitis and infiltrative glioma. It was proposed that the right temporal lesion could represent post-ictal change. MR spectroscopy demonstrated an increased choline peak at the level of the medial left temporal lobe, suggestive of neoplastic disease (see Figure 3). MR perfusion demonstrated patchy areas of hyperperfusion within the left perisylvian region and left anterior temporal lobe, also supporting a diagnosis of neoplastic disease. 

**Figure 4:**
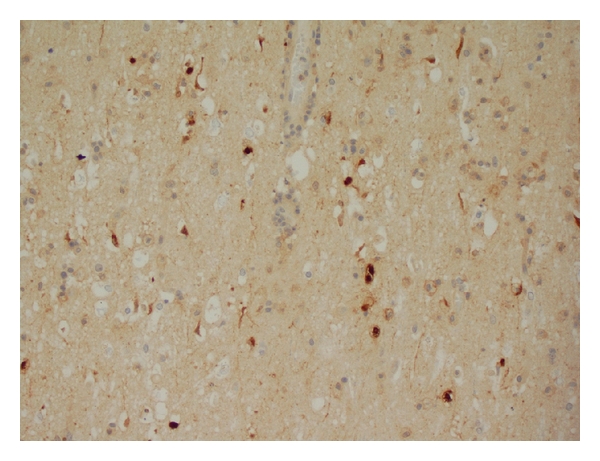
Pathology: HSV positive nuclei.

One week after presentation, patient underwent left temporal craniotomy for open-brain biopsy. Pathology revealed HSV positive nuclei in the cortex and subcortical white matter (see Figure 4). Intravenous acyclovir was given for a total of 21 days. The patient became more responsive but continued to be disoriented and emotionally labile. She was released to a subacute rehabilitation facility, and a year after her symptoms, she was living at home, independent in her activities of daily living. On cognitive evaluation, the patient had some short-term memory and word comprehension difficulties. As she had no clinical seizures during her hospitalization and ambulatory EEG showed only left temporal lobe slowing, she was weaned off anticonvulsant medication.

## 3. Discussion

The diagnosis of HSE can be clouded by confounding factors especially when CSF cannot be obtained. Our patient had headaches and significant weight loss in the months prior to presentation, supportive of a diagnosis of malignancy. Although CRP and ESR were elevated, these acute-phase proteins are not specific to etiology and may be elevated in response to tumor, stroke, and seizures as well as infection and inflammation. Pronounced elevations in CRP do not usually occur in malignancy alone. However, it is not uncommon for patients with malignancy to have concurrent illness including infection. Therefore, the context of the case is important when interpreting these results. Moreover, our patient was afebrile throughout her hospital course, which is uncommon in HSE.

Temporal lobe involvement is classical for herpes encephalitis, and the abnormal focus can extend to multiple lobes, a pattern that can also be seen with infiltrative tumors. While the appearance of hemorrhage on imaging is typically petechial in HSE, in this case, the appearance of frank hemorrhage on the initial CT was suspicious for malignancy. Only patchy meningeal enhancement was seen although the literature suggests that meningeal enhancement may not be prominent early on in HSE [3]. 

MR spectroscopy is nonspecific and was misleading in this case. Typical MR spectroscopy findings in brain neoplasms include decreased N-acetylaspartate (NAA), a marker of neuronal integrity, increased choline (Cho), representing cell membrane and myelin turnover, and decreased creatine (Cr), which provides inorganic phosphates for ATP production and hence represent energy stores. Nonneoplastic disease processes in the CNS including infection may result in reactive proliferation of cellular elements of the immune system and of glial tissue, producing MR spectroscopy profiles that are indistinguishable from CNS neoplasms due to the neuron destroying nature of those lesions [9]. In looking at the diagnostic accuracy of MR spectroscopy, one study revealed a sensitivity of 0.95 and a specificity of 1, with an accuracy of 0.96, as judged by 4 nonblinded readers asked to distinguish neoplastic from nonneoplastic spectra in 54 patients. When blinded, upon evaluation of 35 untreated patients, the values were 0.88, 0.80, and 0.86, respectively [10]. MR spectroscopy in HSE generally shows decreased NAA/Cr ratios at various time intervals, which could be compatible with neuronal loss. An increased Cho/Cr ratio may be seen and is thought to reflect myelin breakdown [11–14].

This case demonstrates the difficulty of diagnosis in certain acutely ill neurological patients. Here, the diagnosis was between malignancy and HSE. The appropriate workup (including bloodwork, imaging, and MR spectroscopy) and therapy (with dexamethasone and acyclovir) were undertaken. When confounding results occur, we feel that tissue diagnosis is essential to ensure appropriate disease management. In this case, biopsy was performed because of concerns that a lumbar puncture would precipitate central herniation.
